# Determinants of leptospirosis in Sri Lanka: Study Protocol

**DOI:** 10.1186/1471-2334-10-332

**Published:** 2010-11-19

**Authors:** Suneth B Agampodi, Dhanaseela B Nugegoda, Vasanthi Thevanesam

**Affiliations:** 1 Department of Community Medicine, Faculty of Medicine and Allied Sciences, Rajarata University of Sri Lanka; 2 Department of Microbiology, Faculty of Medicine, University of Peradeniya, Sri Lanka

## Abstract

**Background:**

Leptospirosis is becoming a major public health threat in Sri Lanka as well as in other countries. We designed a case control study to determine the factors associated with local transmission of leptospirosis in Sri Lanka, in order to identify major modifiable determinants of leptospirosis. The purpose of this paper is to describe the study protocol in detail prior to the publishing of the study results, so that the readership will be able to understand and interpret the study results effectively.

**Methods:**

A hospital based partially matched case control design is proposed. The study will be conducted in three selected leptospirosis endemic districts in central Sri Lanka. Case selection will include screening all acute fever patients admitted to selected wards to select probable cases of leptospirosis and case confirmation using an array of standard laboratory criteria. Age and sex matched group of acute fever patients with other confirmed diagnosis will be used as controls. Case to control ratio will be 1:2. A minimum sample of 144 cases is required to detect 20% exposure with 95% two sided confidence level and 80% power. A pre tested interviewer administered structured questionnaire will be used to collect data from participants. Variables included in the proposed study will be evaluated using conceptual hierarch of variables in three levels; Exposure variables as proximal; reservoir and environmental variables as intermediate; socio-demographic variables as distal. This conceptual hierarch hypothesised that the distal and intermediate variables are mediated through the proximal variables but not directly. A logistic regression model will be used to analyse the probable determinants of leptospirosis. This model will evaluate the effect of same level and upper level variables on the outcome leptospirosis, using three blocks.

**Discussion:**

The present national control programme of leptospirosis is hampered by lack of baseline data on leptospirosis disease transmission. The present study will be able to provide these essential information for formulation of better control strategies.

## Background

Leptospirosis is thought to be the most widespread zoonotic disease in the world[1]. It poses a major public health threat to the developing and the developed world as an emerging infectious disease. Tens of millions of people are estimated to be affected annually,[2] resulting in 350,000 to 500,000 cases of severe disease[3]. The disease is endemic in humid, tropical, and subtropical areas of the world where most of the developing countries are located[4]. In Asia Pacific region, Latin America and in Southeast Asia, it is highly prevalent[5] and there has been a marked increase in the number of outbreaks and cases reported during the last two decades. Even though the disease is mostly endemic in rural settings,[6] an increasing number of cases and frequent outbreaks among urban dwellers[7,8] is a recent finding worldwide.

The first confirmed case of leptospirosis was reported in Sri Lanka in 1959[9]. Since then, series of confirmed cases were reported from Gampaha, Kegalle, Ratnapura, and Colombo districts during the 1960's and 70's. Disease notification data shows a steady increase in reporting of leptospirosis over the last two decades in Sri Lanka[10]. This may be either due to emerging disease or due to improved surveillance or both. In 2007, clustering of unidentified fever cases and few deaths were reported from Matara, Gampaha and Kandy district and some of these cases were later confirmed as leptospirosis. During 2008, Sri Lanka experienced the largest ever outbreak of leptospirosis in its history with 7406 reported cases[11].

Despite the fact that the Epidemiology Unit of Sri Lanka predicted the 2008 outbreak correctly, the public health system was unable to control the massive outbreak due to a scarcity of data. Previously, we reviewed leptospirosis in Sri Lanka and explained the need for confirmation of this outbreak through laboratory confirmation and the importance of investigating this probable leptospirosis outbreak[12]. However, studies on the epidemiology and determinants of local disease transmission are lacking, whereas clinical manifestations are discussed in several papers[13-15].

The global literature contains an extensive data base on the epidemiology of leptospirosis. Most of these studies are retrospective, cross sectional descriptive analysis of cases presenting during a specified time frame. Analytical studies provide the best epidemiological tools in determination and quantifying risk associations. Case control studies are used in this type of diseases, because they are cheap, rapid and easy to conduct. Cohort studies are unrealistic because of very low incidence rates necessitating a very large study sample, which is not cost effective. Analyses of risk factors of leptospirosis have been attempted using case control methodology in several studies[16-25]. Retrospective and prospective studies are available and some studies are based on cases identified through cross sectional antibody prevalence studies. Summary of published case control studies of leptospirosis and identified risk factors are summarized in Table 1.

**Table 1:** Risk factors for human leptospirosis based on case control studies

Authors	Country	Study	Sample size	Risk factors	Odds ratio(CI)
Douglin et al (1997)	St. Andrew Barbados	Laboratory based retrospective	Cases 22	Gardening	4.57(1.09-20.36)
			Con. 38	presence of dogs around the home	7.82(1.79-46.55)
				wearing boots in the garden or yard	8.59(1.93-42.55)
				walking through ponds or stagnant water	25.62(2.89-1151.84)
Bovet et al (1999)	Seychellus	Population based prospective	Cases 75	Gardening	9.86 (2.6-36.1)
			Con. 65	Indoor occupation	0.28 (0.09-0.85)
				Home built with corrugated iron	4.6 (1.09-19.4)
				Wet soil around home	5.65 (1.39-23)
				Refuse not collected by public service	5.23 (1.37-20)
				Cats at home	7.55 (2.04-27.9)
				Skin wounds	6.66 (2.04-27.9)
				Drinking locally made brew	5.41 (1.38-21.2)
Leal-Castellanos et al (2003)	Chiapas, Mexico	Rural community prevalence study	1169 subjects	skin cut or abrasion	4.2 (3.1-5.7)
				contact with animal excreta with no	1.9 (1.3-2.7)
				protection and with a skin cut or abrasion	2.3 (1.1-4.6)
Phraisuwan et al (2002)	Thailand	High risk exposure - after pond cleaning	Cases 43	wearing long pants or skirts	0.217
			Con. 61	presence of more than two wounds on the body	3.97
Ashford et al (2000)	Nicaragua	High risk exposure Following an Outbreak	Case 85	Rural household	2.61 (1.06-6.45)
			Con. 481	Gathering wood	2.08 (1.14-3.79)
				Shelling/husking corn	1.8 (0.72-4.51)
				Indoor water source	0.42 (0.22-0.80)
Everard et al	Barbados	Laboratory based retrospective		Sugar-cane workers	5
(1990)				those whose families minded livestock	2.5
				rodents in their garden/yard	1.8
Johnson et al (2004)	Peru	Endemic area seroprevalence	Case 235 Con. 1116	Not wearing shoes in the field	2.17 (1.39-3.37)
Tangkanakul et al (2000)	North- eastern, Thailand	Hospital based Prospective	Case 56	travel on potholed roads	5.0 ( 1.2-20.2)
			Con. 145	traveling by car	0.2 ( 0.06-0.9)
Sarkar et al (2002)	Salvador, Brazil	During an epidemic retrospective population based	Case 101	Open sewer in proximity	5.07 (2.04-12.64)
			Con. 125	Open sewer floods during rainy season	4.21 (1.51-12.83)
				Street floods during rainy season	2.54 (1.08-6.17)
				> 6 h/day outdoors	2.42 (1.16-5.00)
				Contact with sewer water	3.63 (1.69-7.25)
				Contact with floodwater	3.03 (1.44-6.39)
				Contact with mud	3.08 (1.32-5.87)
				Sighting groups of five or more rats	5.00 (2.22-21.25)
				Peri-domiciliar sighting of rats Sighting	3.40 (1.74-11.78)
				Sighting rats at work site	2.40 (1.11-5.17)
				Dog as domestic animal	1.19 (0.57-2.47)
				Works > 40 h/week	1.72 (0.89-3.66)
				Works outdoors exclusively	2.46 (1.04-5.11)
				Work-related contact with trash	2.36 (1.23-5.56)
Nardone et al (1998)	Metropolitan France	Retrospective, hospital-based	Case 90	Skin lesion	7.0 (2.7-17.6)
			Con. 169	Countryside residence	2.9 (1.1-7.6)
				Canoeing	15.5 (1.6-147.0)
				Any animal contact	4.8 (1.4-16.2)

As leptospirosis is becoming a major public health threat in Sri Lanka, and data on local dynamics of disease transmission is lacking, we designed a case control study to determine the factors associated with local transmission of leptospirosis in Sri Lanka. The study protocol was reviewed and approved by the Board of Study in Community Medicine, Post Graduate Institute of Medicine, Sri Lanka and Ethical Review Committee, Faculty of Medicine, University of Peradeniya, Sri Lanka. The study was conducted during the peak of the 2008 outbreak. The purpose of this paper is to describe the study protocol in detail prior to the publishing of the study results, so that the readership will be able to understand and interpret the study results effectively.

## Methods

### Study design

This is a hospital based case control study.

### Study settings

**Figure 1:**
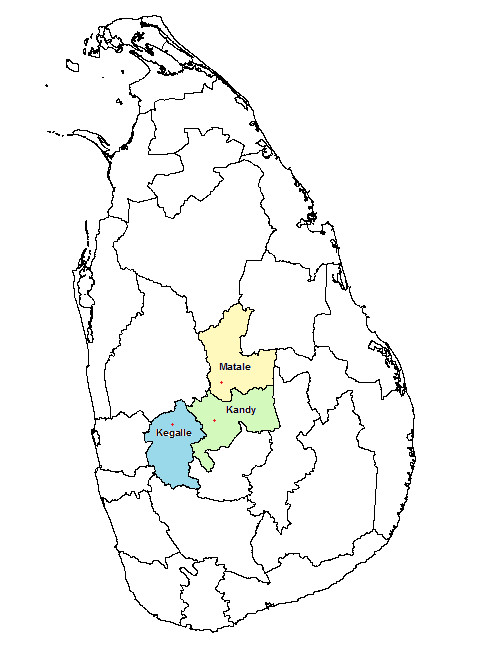
Map of Sri Lanka showing the study area.

This study will be conducted in the districts of Kegalle, Kandy, and Matale, in Sri Lanka. These three districts are adjoining districts in the central part of the country (Figure 1). The Kegalle district is situated in the Sabaragamuwa province and the other two districts are in the Central province. All three districts have been declared as leptospirosis endemic districts in Sri Lanka. Some selected demographic and socio-economic indicators of these three districts are listed in Table 2.

**Table 2:** Selected demographic and socio-economic characteristics of the study settings

	Kandy	Kegalle	Matale
Total population	1,279,028	785,524	441,328
Land area (km^2^)	1,940	1,693	1,993
Crude birth rate (per 1000 population)	22.4	11.5	16.7
Crude death rate (per 1000 population)	6.6	5.8	5
Median per capita income (Rupees)	16,203	13,114	14,119
Paddy harvested area (hectares)	30,752	25,070	14,598

According to routinely reported data, the Kegalle district had one of the highest incidence of leptospirosis in Sri Lanka during the last five years. Kandy and Matale districts show steadily increasing number of reported cases during the same period which was also seen during the present study period. All three districts reported the highest number of cases in the history of Sri Lanka during 2008. Matale district had the highest incidence of leptospirosis in Sri Lanka during the 2008 outbreak.

Three main hospitals in these three selected districts were selected for the present study. All three hospitals are tertiary care units and sentinel sites selected for leptospirosis surveillance by the Epidemiology Unit. Hospitals selected for the present study are listed below.

1. District General Hospital-Kegalle (DGHK)

2. Teaching hospital - Kandy (THK)

3. District General Hospital - Matale (DGHM)

### Study population

The study population will include all clinically suspected leptospirosis patients who will be admitted to the medical wards in the selected government hospitals during the study period. Patients in the paediatric age group will not be included because the study needs invasive procedure (acute and convalescent venous blood sampling) with follow-up which will be difficult for children as well as for parents.. In addition, data on exposure, which will be the main determinant, will be difficult to obtain from paediatric age group. Obtaining data from parents on exposure will not be reliable and comparable to adult patients.

### Study sample

#### Cases

Cases for the present study will be selected after a screening process and confirmatory tests. Recruiting cases will be done prospectively. All fever cases admitted to selected wards will initially be screened using the following inclusion criteria.

1. Patients admitted to medical wards in the selected hospitals

2. Presenting complaint - acute febrile illness (fever less than 15 days and temperature > 37.8°C)

3. One of the following major symptoms:

• Headache

• Myalgia

• Prostration

4. Associated with any of the following signs (at least one):

• Conjunctival suffusion/conjunctival haemorrhage

• Meningeal irritation

• Anuria or oliguria/proteinuria/haematuria

• Jaundice

• Haemorrhages

• Purpuric skin rash

• Cardiac arrhythmia or failure

These inclusion criteria were formulated after an extensive review of literature. The surveillance case definitions used in Sri Lanka by the national surveillance programme[10] as well as case definitions used in other countries were taken into consideration [26-37]. However, the suggested case definitions in other countries and the surveillance case definition in Sri Lanka seemed too stringent for the initial screening. Hence, the case definitions were modified with the help of two experts (A clinician and microbiologist) to increase the yield of the screening, so that mild to moderate cases would also be included in the selected study sample.

The second step of case selection will be the confirmation of screen positives. In Sri Lanka, recommended leptospirosis diagnostic facilities are not available. The available genus specific MAT performed at the Medical Research Institute (MRI), Colombo is recommended when no other options are available. This genus specific test uses the *patoc *strain, which belongs to a saprophytic, non-pathogenic Leptospira *sp*. This is a highly non-specific test and is not included in the laboratory criteria for leptospirosis diagnosis. Disease confirmation of all the selected cases based on the gold standard criteria (MAT) will not be possible in the present study due to severe constraint of funds, logistic difficulties and non-compliance of taking follow-up samples. A confirmed case is defined according to the following criteria.

1. Sero-conversion or a significant increase in *Leptospira sp*. agglutination titre;

a. a MAT sero-conversion: negative first sample and a titre of more than 1:100 in the second sample

b. 4-fold rise in titre between acute and convalescent phase samples; OR

2. A single high *Leptospira *MAT titre greater than or equal to 800; OR

3. Positive PCR; OR

4. A clinical case fulfilling stringent surveillance case definition (surveillance case definition proposed by WHO) and having a positive ELISA IgM test

#### Exclusion criteria

When the patient is critically ill and the accompanying persons are unable to provide these details, those cases will be excluded from the cases.

### Controls

#### Inclusion criteria

Patients admitted to the same wards with a history of fever, but with a confirmed diagnosis of diseases other than leptospirosis will be selected as controls.

1. Patients admitted to medical wards in selected hospitals

2. Presenting complaint - acute febrile illness (fever less than 15 days)

3. Symptoms and signs not suggestive of leptospirosis (not fulfilling the inclusion criteria as probable cases during the first clinical screening)

4. Exclude leptospirosis by ELISA as described in case definition

5. A laboratory confirmed diagnosis other than leptospirosis as a cause for fever

#### Exclusion criteria

1. Critically ill patients who were unable to provide a good exposure history

2. Patient whose diagnosis was ambiguous

For each case, two age and sex matched controls will be selected. Age matching will be done within the range of plus or minus five years.

### Sample size

Sample size was calculated to detect association between leptospirosis and exposure with 95% two sided confidence level and 80% power. The formula suggested by Fless and used in Open Epi, open source epidemiologic statistical software for public health was used to calculate the sample size.

Out of the variables selected, sample size calculation was based on proximate variables because the conceptual framework hypothesized that distal variables are mediated through proximate variables (see the variables). The lowest exposure rates among exposure variables in normal population were estimated as 10% and the exposure in the cases were estimated as 20%.

Based on these assumptions, the minimum sample size required was 144 for each group. With adjustment for probable non-respondent rate of 10%, the final sample size required for the study was 158 cases and 316 controls.

### Data collectors and training of data collectors

Data collectors will include PI and MBBS qualified trained pre-intern medical officer. Recruitment of the data collector was done after a screening process and an interview. The selected data collector will undergo a two-day training which had the following general objectives.

Develop specific and general qualities for the role of data collectors

Be made familiar with the study purpose, procedures, and design

This training will thoroughly discuss the entire study process from pre-data collection activities, actual data collection and post data collection activities. The Data collector will be given a comprehensive knowledge of the entire study process.

### Data collection instruments

An interviewer-administered questionnaire will be used to collect data on co-determinants of human leptospirosis. The questionnaire was formulated in English with a translation to Sinhalese. Re-translation of the questionnaire was done to examine the validity of the translated version.

### Variables

#### Conceptual hierarchical framework of variable categories

Variables included in the study instrument were identified through extensive literature review and formal discussions with epidemiologists, microbiologists and clinicians involved in leptospirosis disease prevention, control and management.

**Figure 2:**
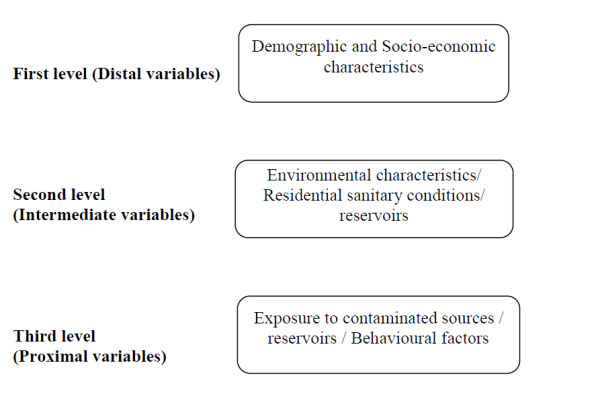
Hierarchical framework of probable determinants of leptospirosis.

The study is planned to evaluate the effects of the postulated risk factors for human leptospirosis among hospital admitted patients. These determinants were chosen after an extensive literature review. The selected determinants were grouped into hierarchical categories according to the conceptual framework for determinants of disease causation[38]. This categorization assumes that each set of distal variable influences the level below or the same level and the effect of the distal variable on the disease is mediated through the proximate variables. This conceptual model implies that demographic and socio-economic variables may determine all variables being studied (Figure 2). Further, it is assumed that only the distal or parallel variables can confound the effect of each variable. Since, the effects of distal variables are mediated through proximate variables; lower level variables cannot be a confounder for upper level variables.

Factors that can be modifiable and those that can be used for preventive and control strategies were included in the list.

#### Outcome measure

The outcome measure will be the odds ratio associated with various exposure risk factors.

#### Pre-testing of the study instrument

Pre-testing of the study instrument will be carried out in order to,

• Determine the time length of the interview

• Improve the wording of the questions

• Eliminate unnecessary questions and add new questions

• Test question sequence

• Correct and improve translation

• Identify conceptually vague items

• Formulate or interview aids to facilitate better interviewing

• Check accuracy and adequacy of questionnaire instructions

• Identify interviewers recoding difficulties.

#### Quality assurance measures

The quality assurance measures will be directed at controlling bias, the interview technique, the preparation of the fieldwork, the conduct of the study, and finally at the plausibility of the database. Constant and close supervision of data collection will ensure the quality of data. More than 50% of data collection will be done by direct supervision of the principal investigator. The PI will visit each centre once in three days for direct supervision and during this visit; spot-checking and back checking will be carried out.

After each interview, each data collector will go over the questionnaire and check for consistency, accuracy and completeness of data. Consistency checks will be run during the data entry to improve the quality of data. Range checks as well as skip and fill applications are incorporated in to data entry programme to ensure that encoded responses are within defined limits.

#### Control of bias

Bias and confounding factors can affect any observational study, especially in case control studies. Several potential sources of biases were considered and measures will be taken to minimize those.

#### Recall bias

Recall bias is the differential recollection of exposure between cases and controls. Study participants in the present study will be patients from the same wards. Laboratory confirmation of the disease status will not be available for patients at the time of data collection which will minimise recall bias in the study. Use of protocols and probing questions and training of data collector will also be done to minimise this bias.

#### Selection bias

Several forms of selection bias may occur in this type of hospital based case control studies. Since the study will be conducted during an epidemic period and all medical practitioners are expected to be aware of the paddy field exposure and leptospirosis, there is a possibility that more patients with this specific exposure will be admitted to the hospital. It might overestimate the final effect size. However, selection of fever patients rather than other patients of normal population as controls will minimise this bias. Discussions will be held with all clinicians and OPD doctors and the eligibility criteria will be provided to them to reduce this bias to a minimal level. Selection bias due to hospital selection will occur if the controls are to be selected from the primary study base (general population). It has been suggested that for hospital based case control studies, using controls with similar manifestations will minimise the selection bias [39-41] and present study will follow these guidelines to minimise selection bias.

#### Interviewer bias

Interviewer bias is the differential collection of exposure data from cases and controls by the interviewer. To minimise this bias only two interviewers will be used in this study. Both interviewers in the study will not be aware of the final diagnosis at the time of data collection. The clinical picture of study participants' being similar would minimize interviewer bias with the control group. In addition, to minimise this effect, study protocols and highly objective close ended questions will be used in the study.

#### Confounding factors

Potential confounding factors for this study include those known socio-demographic factors and environmental factors. Some of the confounding factors will be minimised in the present study using restriction of the study population to adults admitted to particular hospitals. Matching of age and sex for controls will minimise the confounding effect of these known confounders. Details of the other potential confounders will be derived from the questionnaire and multivariate analysis will be used to overcome the effect of these confounding factors.

#### Quality of data

To improve the quality of data, following steps will be taken.

• Blinding of the interviewers and patients on the serological diagnosis

• Use of objectively oriented close ended questions as much as possible

• Selecting incidence cases

• Training of data collectors

• Development of protocols and guidelines for data collection and proper supervision by PI

#### Data processing and analysis

Data will be managed and analyzed using Epi-Info (version 6.04: Centres for Disease Control and Prevention, Atlanta, GA, USA) and Statistical Package for Social Sciences (version 13.0: SPSS Inc., Chicago, IL, USA) respectively.

All open-ended questions will be coded manually. A codebook is prepared for all open and close-ended questions in the study. The codebook contain the variable names and their labels, the response categories and their labels and codes for missing values. Data will be entered manually as a double entry. During data entry, automatic plausibility controls will be conducted to assure quality of data entry. Descriptive statistics will be computed for demographic variables for both cases and controls.

Analysis of determinants will be done according to the conceptual framework. Bivariate analysis of all determinants and unadjusted odds ratios will be calculated first to provide an overall idea about the probable determinants. In the multivariate analysis, three models will be used. In the first model, proximal variables will be entered to the simple logistic regression model to assess the overall effect of these variables. Odds ratios (ORs) and their corresponding 95% confidence intervals will be calculated for these variables. In the next model, intermediate level variables will be entered to the model and then proximal level variables will be added to evaluate confounding effects of the intermediate level variables on proximal variables. Remaining effects of proximal level variables that is not mediated through intermediate level variables will be reflected by corresponding OR for proximal level variables. Risk factors significantly (P < 0.1) associated with outcome on each level will be selected for inclusion in to next level. The confounding effects will be detected through change in OR before and after the adjustment for confounding variable. Final set of risk factors are not the results from the full model, which includes all variables, but from the equation corresponding to the level in which the variable is first entered. This will avoid the possibility that the more proximal determinants will remove the explanatory power of more distal determinants. Adjusted ORs with a p value of < 0.05 will be interpreted as statistically significant association and p value > 0.05 and < 0.1 will be interpreted as having a trend for association.

#### Ethical considerations

Each potential respondent will be first provided with an explanation of the purpose, nature time commitment and potential benefits involved in participating in the study, and will be given an assurance of confidentiality. Each prospective participant will be given an opportunity to ask any question regarding the study prior to recruitment and during the study. Participants will be provided with name and address of the principal investigator to contact him any time during the study. Each potential respondent will be free to decline participation and/or refuse to answer any specific question without any loss of health care benefits or services. Informed written consent will be obtained from all the patients prior to recruitment.

All data collected in this study will be held in the strictest confidence at all levels. Only the PI will have access to collected data and data sets will bee stored in a secure, password protected computer. The personal data that will be collected will be used for only one purpose; to send investigation reports to the patients.

Ethical clearance for the present study was obtained from the Ethical Review Board of the Faculty of Medicine, University of Peradeniya, Sri Lanka.

## Results

Results of this study will be published in early 2011.

## Discussion

The present situation of leptospirosis in Sri Lanka is a major concern among public health professionals as well as clinicians. Hence, the need for a national programme of control and prevention of leptospirosis is urgent. However, the preventive and control measures in Sri Lanka are hampered by the lack of those essential baseline data to understand leptospirosis disease dynamics in the population To decide on proper control and preventive measures determinants of local leptospirosis transmission should be clearly defined. During outbreaks, real time data collection is needed for better understanding of disease epidemiology. We discussed here the rationale for the case control study on leptospirosis and the details of the study protocol. If the determinants of leptospirosis could be clearly identified through this study, it will contribute greatly to control and prevention program of leptospirosis in Sri Lanka.

## Competing interests

The authors declare that they have no competing interests.

## Authors' contributions

Principal responsibility for the study design, conduct, analysis, interpretation and manuscript preparation was assumed by ASB (MD Community Medicine candidate). NDB, and VT participated in design, supervised the conduct, analysis and interpretation, commented on draft and approved the final protocol.

## Pre-publication history

The pre-publication history for this paper can be accessed here:

http://www.biomedcentral.com/1471-2334/10/332/prepub
